# Calcimimetics in CKD—results from recent clinical studies

**DOI:** 10.1007/s00467-008-0900-4

**Published:** 2008-10-01

**Authors:** Georg Schlieper, Jürgen Floege

**Affiliations:** Department of Nephrology and Clinical Immunology, RWTH University Hospital Aachen, Pauwelsstr. 30, 52074 Aachen, Germany

**Keywords:** Calcimimetics, Cinacalcet, Chronic kidney disease, End-stage renal disease, Pediatric patients, Review, Secondary hyperparathyroidism

## Abstract

Secondary hyperparathyroidism (sHPT) is a frequent complication in patients with chronic kidney disease (CKD) and a known contributor to the development of vascular calcification and renal osteodystrophy (CKD–BMD). Secondary hyperparathyroidism is also related to increased cardiovascular mortality in CKD patients. With the discovery that molecules can modulate the calcium-sensing receptor (CaR) of the parathyroid gland, new treatment options are now available to control sHPT. Calcimimetics activate the CaR and—by increasing its sensitivity to calcium—can effectively decrease parathyroid hormone (PTH) secretion. Calcimimetic treatment with cinacalcet has resulted in an effective lowering of PTH levels in both animal and clinical studies. Most clinical studies have been performed in dialysis patients, and only a few studies have been carried out in patients with CKD stage 3 & 4 and renal transplant patients. In haemodialysis patients with sHPT, cinacalcet treatment could increase the number of patients achieving National Kidney Foundation Kidney Disease Outcomes Quality Initiative targets (PTH, calcium, phosphate) compared to standard therapy. In stage 3 and 4 CKD patients, cinacalcet has been reported to reduce PTH levels, however, at the expense of increasing phosphate serum levels. Several small studies have reported that calcimimetics reduced PTH levels and hypercalcaemia after renal transplantation. In addition, two studies on paediatric dialysis patients with sHPT reported effective PTH lowering. This review summarizes recent clinical studies with cinacalcet treatment in CKD patients.

## Background

**Fig. 1:**
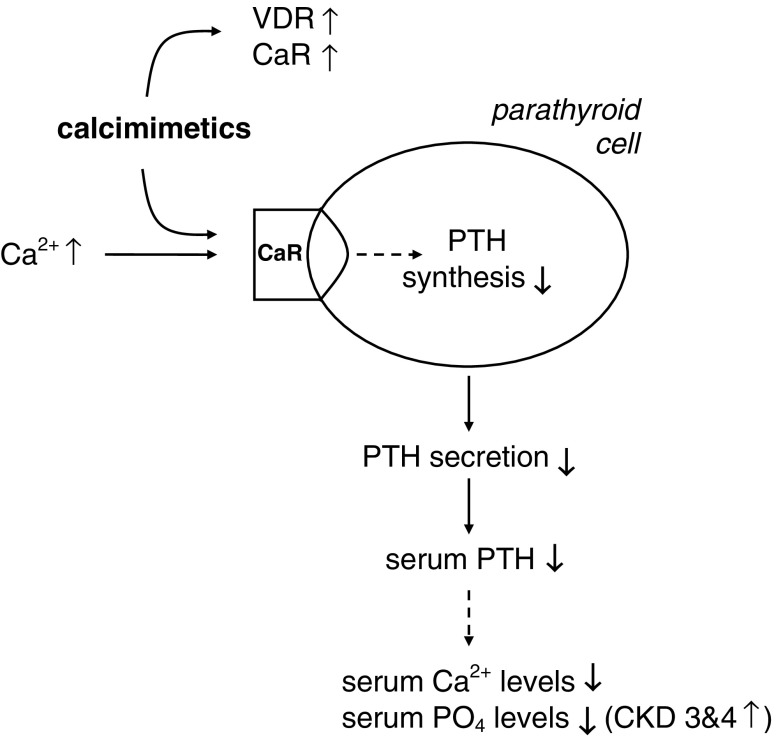
Calcimimetics act on the calcium-sensing receptor (*CaR*) by mimicking and/or potentiating the effects of calcium. Increased serum calcium (*Ca*^*2+*^) levels activate the CaR of the parathyroid cell, which leads—via intracellular signalling mechanisms—to decreased parathyroid hormone (*PTH*) synthesis and secretion. Calcimimetics potentiate the effect of serum calcium and thus can lower serum calcium and serum phosphate (*PO*_*4*_) levels in dialysis patients with secondary hyperparathyroidism. Note that calcimimetics can decrease serum phosphate levels only in dialysis patients but not in patients with stage 3 and 4 chronic kidney disease (*CKD 3&4*) or renal transplant patients. There is also experimental evidence that calcimimetics can increase the numbers of both the CaR and vitamin D receptors (*VDR*) in parathyroid cells

Secondary hyperparathyroidism (sHPT) frequently develops during stages 3 and 4 of chronic kidney disease (CKD3/4). Increased serum levels of intact parathyroid hormone (PTH) have been associated with cardiovascular calcifications, morbidity and mortality in dialysis patients. Prolonged hypocalcaemia, hyperphosphataemia, low calcitriol and elevated FGF-23 levels all contribute to increased PTH synthesis and secretion. Phosphate retention as well as increased FGF-23 levels inhibit the activity of the 1α-hydroxylase, which contributes to decreased calcitriol synthesis and constitutes an additional indirect mechanism by which they feed into elevated PTH synthesis. Moreover, the numbers of calcium-sensing receptors (CaR) and vitamin D receptors (VDR) are lower in CKD patients, resulting in a decreased sensitivity of the parathyroid gland to both serum calcium changes and calcitriol. The CaR is responsible for calcium-mediated signalling in parathyroid cells and plays a central role in the regulation of synthesis and secretion of PTH. Calcimimetics activate the CaR by mimicking or potentiating the inhibitory effects of extracellular calcium on parathyroid cells (Fig. 1). This leftward shift in the set point for calcium-regulated PTH secretion by calcimimetics leads to a reduction in PTH secretion. Calcimimetic compounds can be either inorganic (e.g. Mg^2+^) or organic molecules (e.g. spermine) or phenylalkylamine derivatives (e.g. cinacalcet). Traditionally, secondary hyperparathyroidism (sHPT) has been treated with calcium and vitamin D therapy plus phosphate binders; however, with these interventions, only a few patients achieved the targets—especially all of the targets—of the National Kidney Foundation Kidney Disease Outcomes Quality Initiative (KDOQI). Thus, the introduction of cinacalcet as a calcimimetic compound offers new therapeutic options to control sHPT in CKD patients.

## Studies of cinacalcet in dialysis patients with sHPT

### Studies assessing biochemical endpoints

It has been possible to demonstrate the safety and efficacy of cinacalcet treatment of sHPT in dialysis patients in controlled trials despite standard therapy with active vitamin D and phosphate binders as appropriate (Table 1) [1–4]. Of note, cinacalcet treatment did not only result in a marked decrease in serum PTH but also in significant reductions in serum calcium, phosphate and the calcium–phosphate product. A meta-analysis revealed an average reduction in PTH of 290 pg/mL, in serum calcium of 0.85 mg/dL, in serum phosphate of 0.29 mg/dL and in the calcium–phosphate product of 7.90 mg^2^/dL^2^ [5]. The early decrease in serum calcium, phosphate and the calcium–phosphate product may be interpreted as the analogue of a “hungry bone” after parathyroidectomy, where the bone avidly incorporates calcium and phosphate and thus lowers serum calcium and phosphate levels. However, it is notable that in a long-term study, cinacalcet did lead to persistent reductions of the calcium–phosphate product [6] under conditions when a “hungry bone” can no longer be implicated.

**Table 1:** Clinical studies with cinacalcet

Study design	Key results	Reference
Dialysis patients		
18 weeks of 20–50 mg AMG 073 (cinacalcet) for sHPT treatment in 39 haemodialysis patients (vs. 39 placebo); randomized, placebo-controlled, double-blind	Reduction of PTH (−26%), Ca^2+^ (−4.7%), PO_4_ (−7.5%), and Ca × PO_4_ (−11.9%)	Lindberg et al. [1]
18 weeks of 25–100 mg AMG 073 (cinacalcet) for sHPT treatment in 36 haemodialysis patients (vs. 35 placebo); randomized, placebo-controlled, double-blind	Reduction of PTH (−32%), Ca^2+^ (−4.6%), and Ca × PO_4_ (−7.9%)	Quarles et al. [2]
26 weeks of 30–180 mg cinacalcet for sHPT treatment in 371 haemodialysis patients (vs. 370 placebo); randomized, placebo-controlled, double-blind	Reduction of PTH (−43%), Ca^2+^ (−6.8%), PO_4_ (−8.4%), and Ca × PO_4_ (−14.6%)	Block et al. [3]
26 weeks of 30–180 mg cinacalcet for sHPT treatment in 395 dialysis patients (vs. 101 placebo); randomized, placebo-controlled, double-blind	Reduction of PTH (−40%), Ca^2+^ (−6.5%), PO_4_ (−7.2%), and Ca × PO_4_ (−12.8%)	Lindberg et al. [4]
Long-term (100 weeks) effect of 30–180 mg cinacalcet in 59 haemodialysis patients with sHPT; open-label, single-arm	Reduction of PTH (−24%)	Moe et al. [6]
Retrospective analysis of three placebo-controlled, double-blind 26-week studies (665 dialysis patients with 30–180 mg cinacalcet vs. 471 placebo)	Achievement of PTH target below 300 pg/mL and KDOQI targets (cinacalcet vs. control): PTH (56 vs. 10%), Ca^2+^ (49 vs. 24%), PO_4_ (46 vs. 33%), Ca × PO_4_ (65 vs. 36%)	Moe et al. [7]
Retrospective analysis of outcome parameters in four double-blind randomized studies in 697 dialysis patients with sHPT and cinacalcet plus standard therapy over 12 months (vs. 487 standard therapy only)	Reduction of parathyroidectomy rate (−93%), fractures (−54%) and cardiovascular hospitalization (−39%)	Cunningham et al. [14]
Meta-analysis of eight randomized, double-blind cinacalcet vs. placebo trials in 737 dialysis patients (vs. 541 placebo-treated patients)	Reduction of PTH (−290.49 pg/mL), Ca^2+^ (−0.85 mg/dL), PO_4_ (−0.29 mg/dL), and Ca × PO_4_ (−7.90 mg^2^/dL^2^)	Strippoli et al. [5]
4 hours after 30–120 mg cinacalcet in 20 dialysis patients with sHPT (vs. 12 controls); open-label, comparative study	Increase of substance P and decrease of vasoactive intestinal peptide (VIP)	Diez et al. [13]
23 weeks of cinacalcet in 368 haemodialysis patients with sHPT (184 controls) (OPTIMA); randomized, open-label	Achievement of PTH target below 300 pg/mL (71% vs. 22%)	Messa et al. [8]
14 weeks of 25–100 mg cinacalcet in 72 Japanese haemodialysis patients with sHPT (vs. 71 placebo); randomized, placebo-controlled, double-blind	Reduction of PTH (−54%), Ca^2+^ (-9%), PO_4_ (−10%), and Ca × PO_4_ (−18%); reduction of osteocalcin (−37%) and tartrate-resistant acid phosphatise (−16%)	Fukagawa et al. [9]
16 weeks of cinacalcet plus low-dose vitamin D (TARGET) for treatment of sHPT in 444 haemodialysis patients; open-label, single-arm	Reduction of PTH (−35%), Ca^2+^ (−11%), PO_4_ (−7%), and Ca × PO_4_ (−17%).	Block et al. [11]
3 months of 30–120 mg cinacalcet in nine paediatric dialysis patients with sHPT; open-label, single-arm	Reduction of PTH (−52%) and alkaline phosphatase (−31%)	Silverstein et al. [16]
4 weeks of 0.25 mg/kg body weight cinacalcet in seven paediatric patients with sHPT; open-label, single-arm	Reduction of PTH (−79%), Ca^2+^ (−7%), PO_4_ (−33%), and Ca × PO_4_ (−41%).	Muscheites et al. [17]
12 months of cinacalcet in an European study (ECHO) with 1852 haemodialysis patients with sHPT; open-label, single-arm	Reduction of PTH (−49%), and Ca × PO_4_ (−17%)	Vervloet et al. [10]
27 weeks of titrated cinacalcet plus low-dose vitamin D (*n* = 87) vs. vitamin D alone (*n* = 86) in dialysis patients (ACHIEVE) with sHPT; randomized, controlled, open-label	Better achievement of PTH target below 300 pg/mL	Fishbane et al. [12]
2.5 to 4 years of cinacalcet in 1900 haemodialyis patients (vs. 1900 placebo) with sHPT (EVOLVE); prospective, randomized, placebo-controlled, double-blind	Ongoing study. Primary outcome: combination of death and cardiovascular events	Chertow et al. [15]
52 weeks of cinacalcet plus standard therapy in 165 haemodialysis patients (vs. 165 standard therapy only) (ADVANCE); randomized open-label	Ongoing study. Primary outcome: progress of coronary calcifications	
Chronic kidney disease stage 3 and 4 patients		
18 weeks of 30–180 mg cinacalcet in 27 CKD3 and CKD4 patients (vs. 27 placebo) with sHPT; randomized, placebo-controlled, double-blind	Reduction of PTH (−35%), Ca^2+^ (−7%), increase in PO_4_ (10%) and Ca × PO_4_ (7%)	Charytan et al. [20]
32 weeks of 30–180 mg cinacalcet in 295 CKD3 and CKD4 patients (vs. 100 placebo) with sHPT; randomized, placebo-controlled, double-blind	Reduction of serum PTH and Ca^2+^, increase of serum PO_4_ and urinary Ca^2+^ excretion	Chonchol et al. [21]
Renal transplant patients		
Review with report on eight small studies with 3–18 months cinacalcet treatment in 83 renal transplant patients	Reduction of PTH and Ca^2+^, mostly increase of PO_4_	Chonchol and Wüthrich [23]
One week of 30 mg cinacalcet in 14 renal transplant patients; open-label, single-arm	Decrease of serum tacrolimus level by 14%, increase of the cylcosporin metabolite AM19 by 9%	Falck et al. [24]

*sPTH* Secondary hyperparathyroidism; *PTH* parathyroid hormone; *CKD3, 4* chronic kidney disease stage 3, 4

A retrospective analysis could demonstrate that cinacalcet treatment increased the percentage of patients achieving the KDOQI targets [7]. In this study, data of three placebo-controlled, double-blind, 26-week studies with similar design were combined. A total of 1136 subjects on dialysis were randomized to receive traditional therapy plus cinacalcet or placebo. Cinacalcet intake was titrated from 30 mg to 180 mg per day. The number of patients achieving both KDOQI targets for phosphate and calcium and PTH levels below 300 pg/mL increased significantly from 6 to 41% [7]. The OPTIMA study confirmed these data in a prospective fashion [8]. In this latter study, 552 patients were randomized to conventional treatment or cinacalcet. The primary endpoint, i.e. the proportion of patients with mean PTH levels below 300 pg/mL, could be achieved in 71% of the patients in the cinacalcet group versus 22% in the control group [8]. A recent Japanese study also confirmed the effect of cinacalcet on PTH lowering but reported a lower dosage of cinacalcet needed to achieve the PTH target in that Asian population [9]. First results of the ECHO study, a European observational study in 1800 haemodialysis patients with sHPT, confirmed the results of the controlled studies with cinacalcet treatment [10]. After 12 months of treatment with cinacalcet, PTH levels could be reduced by 49% and the calcium–phosphate product by 17%. Again, the dosage of cinacalcet necessary to achieve this appeared to be lower than in the controlled studies, with most patients ingesting 30 or 60 mg cinacalcet daily [10].

Two studies investigated the effects of cinacalcet added to low doses of vitamin D in dialysis patients. In the open-label TARGET study, 444 haemodialyis patients were treated with cinacalcet and around 80% of the patients were on vitamin D treatment, which was reduced in the second part of the study [11]. Cinacalcet plus low-dose vitamin D treatment was able to improve KDOQI targets [11]. Another study investigated the combination of cinacalcet with a fixed low-dose vitamin D therapy versus vitamin D alone (where flexible dosing was allowed) in 173 dialysis patients (ACHIEVE) [12]. In this study, the fixed combination of cinacalcet plus low-dose vitamin D resulted in an effective PTH lowering when compared to vitamin D alone. However, in both of the studies, no direct comparison of cinacalcet without vitamin D versus cinacalcet plus vitamin D was made. Thus, it can only be concluded that cinacalcet plus vitamin D is a feasible therapeutic strategy, but the advantage of vitamin D added to cinacalcet has yet to be proven.

Side effects of cinacalcet are most commonly related to gastrointestinal disturbances and include nausea, vomiting, diarrhoea and loss of appetite. Cinacalcet treatment causes only minimal changes in gut hormones [13]. Whether the decrease in vasoactive intestinal peptide (VIP) or the increase in substance P at 4 h after cinacalcet ingestion is responsible for the unwanted symptoms remains to be determined. Alternatively, these side effects may be related to cinacalcet binding to the calcium-sensing receptor in the gut, i.e. direct cinacalcet effects.

### Studies assessing clinical endpoints

As increased PTH levels are associated with increased mortality, it is of interest to determine whether cinacalcet treatment can improve clinical outcomes. With the aim of assessing the efficacy of cinacalcet treatment, Cunningham et al. [14] performed a retrospective analysis of various intervention studies that primarily looked at biochemical parameters (see above). In this analysis, pooled data from four randomized controlled studies were evaluated, and several outcome parameters were measured in more than 1400 haemodialysis patients at 6–12 months: parathyroidectomy, fractures, hospitalization, mortality and self-reported quality of life. Cinacalcet treatment plus standard care led to a significantly reduced rate of parathyroidectomies (−93%) and fractures (−54%) when compared to the group with standard care alone. While the number of all-cause hospitalizations did not differ between the two groups, patients in the cinacalcet group had significantly reduced cardiovascular (CV)-related hospitalizations (−39%). However, this benefit became most notable during the later, open-label phase of the studies and when patient numbers had dropped markedly. This outcome thus represents an interesting pilot observation, which will require confirmation in adequate prospective studies. In terms of quality of life, patients randomized to cinacalcet treatment reported an improvement in physical function but not in the other domains [14]. Finally, over the course of the short-term observation, there was no significant difference in mortality in the group treated with cinacalcet compared to the standard treatment group.

Following these above pilot studies, the Evaluation of Cinacalcet HCl Therapy to Lower CV Events (EVOLVE) study was initiated. EVOLVE is a randomized placebo-controlled trial investigating the effect of cinacalcet treatment on mortality and CV events [15]. In this world-wide phase 3 randomized, double-blind, controlled clinical trial, approximately 3800 haemodialyis patients with sHPT have been assigned to cinacalcet or placebo in addition to traditional therapy. Recruitment finished in early 2008, and the study, which is event-driven, is expected to last a further 2.5 years. The primary composite endpoint is time to all-cause mortality or the first occurrence of a non-fatal CV event (myocardial ischaemia or infarction, heart failure, or peripheral vascular event). Cinacalcet will be titrated between 30 and 180 mg/day based on PTH concentrations, and flexible use of active vitamin D derivatives and phosphate binders will be permitted in both treatment groups. The aim of a second randomized world-wide trial is to investigate whether cinacalcet can slow down the progression of coronary artery calcification in haemodialysis patients (ADVANCE trial = A randomizeD VAscular calcificatioN study to evaluate the effects of CinacalcEt). In this study, approximately 330 haemodialysis patients will be investigated over the course of 1 year, with patients assigned to either cinacalcet in combination with low-dose vitamin D or standard therapy.

Taken together, cinacalcet has been shown to reduce not only PTH levels effectively but also to lower serum calcium and phosphate levels in CKD patients on dialysis. Whether these changes will lead to reduced morbidity or a survival benefit in dialysis patients is currently under investigation.

## Studies in paediatric dialysis patients with sHPT

As the problem of sHPT is not limited to adult CKD patients, calcimimetics also provide a therapeutic option in the treatment of sHPT in paediatric dialysis patients. Two open-label single-arm studies reported in this issue of the journal investigated cinacalcet treatment in nine and seven paediatric patients with end stage renal disease (ESRD) and sHPT, respectively [16, 17]. The young patients (range 7.5–17.5 and 1.1–19 years, respectively) were both on haemodialysis and peritoneal dialysis regimens. In these studies, cinacalcet treatment resulted in a 61% and 79% decline of serum PTH levels, respectively. This decrease in PTH seems to be more pronounced in paediatric patients when compared to adults (Table 1). The cinacalcet dosage was therefore reduced to 0.25 mg/kg body weight in the second study [17], which is significantly lower that the usual starting dose of 30 mg in adults. It is possible that even lower starting dosages than the suggested 0.25 mg/kg should be considered (e.g. 0.15 mg/kg body weight).

Calcimimetics do have the potential to interfere with growth during adolescence (epiphyseal chondrocytes express the CaR). However, at least one experimental study was unable to find a significant effect of cinacalcet on longitudinal growth in uremic rats [18]. Another concern associated with treating adolescent children with calcimimetics is that lower testosterone levels have been reported recently in adult patients on cinacalcet treatment [19].

Taken together, cinacalcet seems to be equally effective in children as in adults; however, studies investigating long-term consequences of cinacalcet therapy in adolescents need to be performed.

## Studies of cinacalcet in CKD 3 and 4 patients with sHPT

Since sHPT often develops in stages 3 and 4 of CKD, an attractive option is to start cinacalcet treatment in earlier stages with the aim of controlling sHPT effectively before advanced or tertiary hyperparathyroidism develops. One preliminary study reported that cinacalcet can efficiently treat sHPT in CKD patients not receiving dialysis [20]. In this double-blind placebo-controlled study, cinacalcet treatment was evaluated in 54 patients with CKD 3 and 4. Cinacalcet treatment decreased PTH levels by approximately 35% and lowered serum calcium levels by 7%, whereas the levels of serum phosphate and the calcium–phosphate product increased by approximately 10 and 7%, respectively [20]. A larger double-blind randomized study with nearly 400 CKD 3 and 4 patients recently confirmed these results and reported an effective reduction in serum PTH and calcium levels while, again, phosphate levels increased with cinacalcet treatment [21]. Whether such an increase in serum phosphate levels bears a risk for vascular calcification, or other adverse outcomes, remains to be determined.

## Studies of cinacalcet in renal transplant patients with persistent sHPT

Hyperparathyroidism frequently persists after successful renal transplantation; in particular, pronounced phosphaturia with resulting hypophosphataemia are frequent observations early after renal transplantation. Oral or parenteral phosphate replacement in such instances may contribute to intra-graft calcifications, a recently recognized cause of inferior graft survival [22]. Cinacalcet provides an attractive alternative therapeutic option in this latter case, but also for patients with persistent hyperparathyroidism long after transplantation. The results from eight small studies assessing the off-label use of cinacalcet in a maximum of 18 transplant patients were recently summarized by Chonchol and Wüthrich [23]. In all of these studies, relatively low cinacalcet doses were needed (average, 30–40 mg/day) to control hyperparathyroidism. Therapy with cinacalcet led to PTH and serum calcium decreases while phosphate serum levels were mostly upregulated without adversely affecting graft function [23]. As cinacalcet may interact with immunosupressants, a recent study with 14 transplanted patients investigated the effect of cinacalcet on serum levels of cyclosporine, tacrolimus and mycophenolate [24]. Cinacalcet treatment induced a 14% decrease in tacrolimus levels while cyclosporine and mycophenolate levels were not significantly altered. The level of the cyclosporine metabolite AM19 was increased by 9%, and this increase was paralleled by a small but significant reduction in glomerular filtration rate (GFR) [24]. Thus, immunosuppressant levels and graft function need to be monitored closely when cinacalcet treatment has been initiated or altered in transplanted patients.

## Other potential indications for cinacalcet in CKD patients

In addition to the treatment of sHPT, cinacalcet may be used to treat other clinical conditions in CKD patients. The most commonly recommended approach to treating primary hyperparathyroidism in symptomatic patients is parathyroidectomy. Even asymptomatic patients may be considered for parathyroidectomy when they meet criteria, such as young age, increased serum calcium levels, increased urinary calcium excretion, reduced bone density or decreased renal function [25]. In a multicentre, randomized, double-blind, placebo-controlled study with 78 patients, cinacalcet normalized serum calcium and reduced PTH in patients with primary hyperparathyroidism. As these effects were maintained with long-term treatment, cinacalcet may be an effective, non-surgical approach for the management of primary hyperparathyroidism [26]. Very recently, a small study on four patients was able to demonstrate that cinacalcet can lower PTH levels after unsuccessful parathyroidectomy [27]. Another condition for which calcimimetics can be used is parathyroid carcinoma. In patients with inoperable parathyroid carcinoma, cinacalcet was shown to effectively lower serum calcium levels in about two thirds of the patients [28]. In this study with 29 patients, PTH levels could only be slightly decreased; however, this result demonstrates that calcimimetics offer a therapeutic option for calcium lowering in patients with parathyroid carcinoma when other treatment options fail.

Finally, the gastrointestinal, in particular colonic, expression of the CaR may be of therapeutic interest in completely different clinical scenarios. Preliminary evidence shows that the chemopreventive action of calcium and calcitriol in colon cancer is mediated—at least in part—through the CaR [29] and that receptor agonists were able to reverse cholera toxin and shiga toxin-induced secretory diarrhoea [30]. Moreover, it has been shown in animal experiments that calcimimetics may exert beneficial effects on blood pressure in uremia and may ameliorate kidney damage in subtotally nephrectomized rats [31–33]. Thus, in the future, clinical indications for calcimimetics may reach far beyond the control of PTH secretion.
